# Ambient heat and risks of emergency department visits among adults in the United States: time stratified case crossover study

**DOI:** 10.1136/bmj-2021-065653

**Published:** 2021-11-25

**Authors:** Shengzhi Sun, Kate R Weinberger, Amruta Nori-Sarma, Keith R Spangler, Yuantong Sun, Francesca Dominici, Gregory A Wellenius

**Affiliations:** 1 Department of Environmental Health, Boston University School of Public Health, Boston 02118, MA, USA; 2 OptumLabs, Eden Prairie, MN, USA; 3 School of Population and Public Health, University of British Columbia, Vancouver, BC, Canada; 4 Harvard T H Chan School of Public Health, Department of Biostatistics, Boston, MA, USA

## Abstract

**Objective:**

To quantify the association between ambient heat and visits to the emergency department (ED) for any cause and for cause specific conditions in the conterminous United States among adults with health insurance.

**Design:**

Time stratified case crossover analyses with distributed lag non-linear models.

**Setting:**

US nationwide administrative healthcare claims database.

**Participants:**

All commercial and Medicare Advantage beneficiaries (74.2 million) aged 18 years and older between May and September 2010 to 2019.

**Main outcome measures:**

Daily rates of ED visits for any cause, heat related illness, renal disease, cardiovascular disease, respiratory disease, and mental disorders based on discharge diagnosis codes.

**Results:**

21 996 670 ED visits were recorded among adults with health insurance living in 2939 US counties. Days of extreme heat—defined as the 95th centile of the local warm season (May through September) temperature distribution (at 34.4°C *v* 14.9°C national average level)—were associated with a 7.8% (95% confidence interval 7.3% to 8.2%) excess relative risk of ED visits for any cause, 66.3% (60.2% to 72.7%) for heat related illness, 30.4% (23.4% to 37.8%) for renal disease, and 7.9% (5.2% to 10.7%) for mental disorders. Days of extreme heat were associated with an excess absolute risk of ED visits for heat related illness of 24.3 (95% confidence interval 22.9 to 25.7) per 100 000 people at risk per day. Heat was not associated with a higher risk of ED visits for cardiovascular or respiratory diseases. Associations were more pronounced among men and in counties in the north east of the US or with a continental climate.

**Conclusions:**

Among both younger and older adults, days of extreme heat are associated with a higher risk of ED visits for any cause, heat related illness, renal disease, and mental disorders. These results suggest that the adverse health effects of extreme heat are not limited to older adults and carry important implications for the health of adults across the age spectrum.

## Introduction

Exposure to high ambient temperature is recognized as a major threat to public health and is associated with substantial excess morbidity and mortality.^1^ Extreme heat is one of the leading causes of weather related deaths in the United States,^2^ leading to thousands of excess deaths annually.^3^ ^4^ Owing to continued climate change, days of extreme heat are projected to become more frequent and more intense in the future.^5^ Thus, the burden of disease associated with days of extreme heat is already high and expected to increase further.^5^

Although the adverse health impacts of heat on heat related,^6^ ^7^ renal,^6^ cardiorespiratory,^8^ ^9^ and mental^10^ ^11^ ^12^ illnesses are well documented among older adults, less is known about the potential health impacts of heat on young and middle aged adults. The most comprehensive studies of the health effects of heat in US adults have focused on either mortality or hospital admissions among Medicare beneficiaries aged 65 years and older,^8^ ^13^ ^14^ ^15^ ^16^ ^17^ likely owing to the availability of national datasets in this population. Moreover, few studies have assessed the impacts of heat on rates of emergency department (ED) visits on a national scale. ED visits might serve as a more sensitive indicator of the health impacts of heat and a more appropriate signal for syndromic surveillance,^10^ ^18^ particularly among young and middle aged adults. The available evidence indicates that heat also poses a considerable health threat for young and middle aged adults, although results from studies have not been consistent.^6^ ^9^ ^18^ ^19^ ^20^ ^21^ ^22^ ^23^ ^24^ ^25^ ^26^ ^27^ For example, heat in California has been associated with a higher risk of ED admissions for any cause among those aged 5 to 64 years^18^ and in 12 Chinese cities among those aged 15 to 64 years,^25^ but an association was not found in studies in the US states of Rhode Island^6^ and Texas,^27^ or in Sydney, Australia.^26^

In this study we quantified the risk of ED visits for any cause and cause specific conditions associated with a range of temperatures observed during the warm season among adults aged 18 years and older living in the conterminous US. We analyzed healthcare utilization deidentified claims data from the OptumLabs® Data Warehouse^28^ and identified more than 22 million ED visits among people enrolled in commercial and Medicare Advantage health insurance plans and residing in 2939 US counties between May and September 2010 to 2019. First, we examined the associations between heat and rates of ED visits for any cause, heat related illness, renal disease, cardiovascular disease, respiratory disease, and mental disorders. Then we investigated whether observed associations of heat with ED visits for any cause differed across strata defined by age, sex, low income status, climate zone, and geographic region.

## Methods

### Study population

In this study we used deidentified medical claims between 1 May and 31 September 2010 to 2019 from the OptumLabs Data Warehouse,^28^ which includes medical and pharmacy claims, laboratory results, and enrollment records for commercially insured and Medicare Advantage enrollees. The database contains longitudinal health information on enrollees and patients, representing a diverse mixture of ages, ethnicities, and geographical regions across the US. We identified claims for ED visits based on ICD-9 and ICD-10 (international classification of diseases, ninth and 10th revisions, respectively) codes, revenue code, Current Procedural Terminology code, and place of service code (supplementary table S1). For each claim we then extracted information on age, sex, and county of residence of the individual, as well as the admission date and principal diagnosis code (based on ICD-9 or ICD-10). We limited our analysis to ED visits occurring among people aged 18 years and older.

We considered a range of causes for ED visits based on the principal diagnosis code, including any cause (ICD-9: 001-V91 or ICD-10: A00-Z99), heat related illness (ICD-9: 276, 992, E900.0, E900.9 or ICD-10: T67, E86, E87, X30), renal disease (ICD-9: 580-589 or ICD-10: N00-N05, N08, N17-N19, N25-N27), cardiovascular disease (ICD-9: 390-459 or ICD-10: I00-I99), respiratory disease (ICD-9: 460-519 or ICD-10: J00-J99), and mental disorders (ICD-9: 290-319 or ICD-10: F01-F99).^6^ ^29^ Additionally we included ED visits for epilepsy (ICD-9: 345 or ICD-10: G40, G41) as a putative negative outcome control because of the lack of known biological plausibility for the association between heat and epilepsy. We aggregated the daily number of ED visits by age (18-24, 25-34, 35-44, 45-64, 65-74, and ≥75 years); sex (men *v* women); geographic region of the country, as defined by the US Global Change Research Program’s Fourth National Climate Assessment (NCA4)^30^; and climate zone of the country as defined by the Köppen-Geiger Climate Classification system (supplementary figure S1).^31^ Among a subset of members (32% (n=23 803 556/74 188 445) of unique beneficiaries) who were enrolled in Medicare Advantage programmes that include pharmacy benefits, we also aggregated the daily number of ED visits by low income status (yes *v* no), defined by whether or not members qualified for the low income subsidy under the Medicare Part D prescription drug programme.

### Assessment of ambient temperature

**Fig 1:**
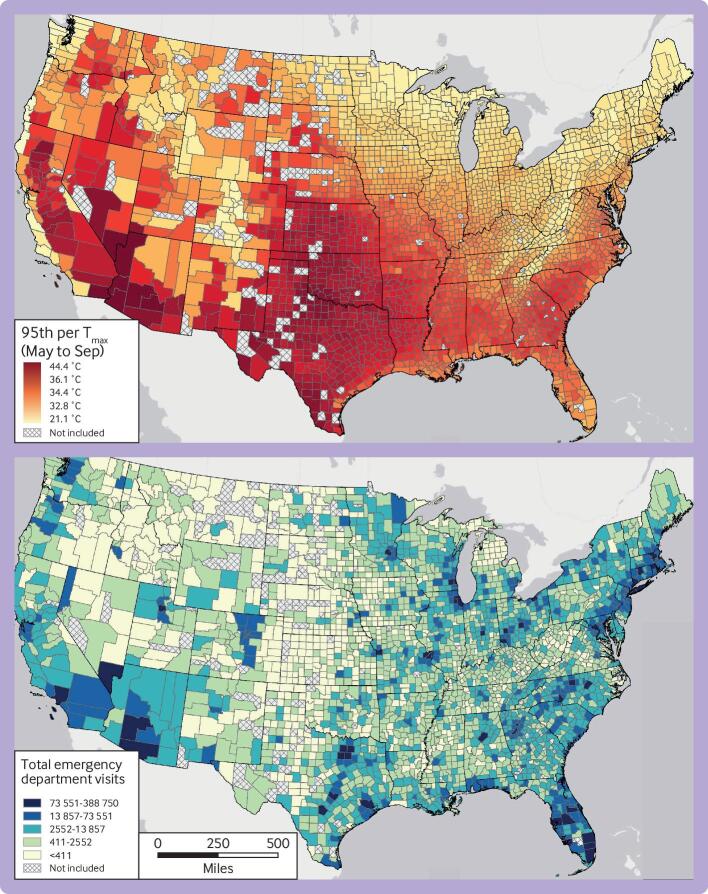
Maps of extreme temperature and number of emergency department visits between May and September 2010-19. (Top panel) Warm season average daily maximum ambient temperature at 95th centile. (Bottom panel) Number of emergency department visits. Counties in gray were not included in the analysis because no emergency department visits were recorded during the study period

Daily maximum ambient temperature was estimated using the Parameter-elevation Relationships on Independent Slopes (PRISM) model, a spatiotemporal model with horizontal grid spacing of about 2.5 miles (4 km).^32^ To represent population exposure to temperature, we calculated a population weighted average of daily maximum temperature for each day in each county, as described previously^33^ and in the supplementary appendix. We used daily maximum ambient temperature during the summer months (May to September; the warm season) to represent heat exposure (fig 1) and calculated temperature centiles by day in each county to reflect county specific distribution of temperature.^34^

### Statistical analysis

We used a χ^2^ test to compare beneficiaries in the health plans on 1 July 2015 versus the 2015 US population for age, sex, and geographic region. To estimate the association between county specific daily maximum temperature centile and all cause and cause specific ED visits for May to September 2010-19, we used a time stratified case crossover design.^35^ ^36^ In this study design, participants serve as their own control, and the inference is based on the comparison of daily ambient temperatures on the case day versus daily ambient temperatures on control days.^37^ Specifically, the case day was defined as the admission date of each ED visit, and control days were selected at the same year and month as the case day to control for seasonal and long term time trends. Control days were other days in the same month and day of week as the case day. This design has the advantage of controlling for potential confounding by all known and unknown individual and county level covariates that do not vary day to day; including, for example, age, sex, race, socioeconomic status, and population density, and behavior risk factors, such as smoking.^35^

We applied a well established distributed lag non-linear modeling framework to allow for both non-linear exposure-response functions and non-linear lag response functions.^38^ As in previous studies,^39^ we modeled exposure-response functions using a quadratic B spline with one internal knot placed at the 50th centile of county specific warm season’s temperature distribution. We modeled the lag response function using a natural cubic B spline with two knots placed at equal intervals on the log scale of lags up to five days. In all models we additionally adjusted for relative humidity and federal holidays. We used conditional logistic regression to estimate odds ratios and 95% confidence intervals of ED visits associated with warm season temperature. To facilitate communication, we defined extreme heat locally in each county as days with a maximum daily temperature equal to the 95th centile of the warm season temperature distribution in that county and report odds ratio for days of extreme heat relative to the temperature of minimum morbidity. We identified the temperature of minimum morbidity based on the temperature centile (bounded between the 1st and 99th centiles) associated with the lowest rate of all cause ED visits based on the overall cumulative exposure-response association.^38^ To more fully characterize the adverse health effects of heat, we also report odds ratio of ED visits associated with days of moderate heat, defined as days when the maximum daily temperature was equal to the 85th centile of the local distribution of temperatures during the warm season.

Results are expressed in terms of both the excess relative risk and the excess absolute risk of ED visits associated with heat. Excess relative risk was defined as (odds ratio−1)×100%, and excess absolute risk was defined as α×(odds ratio−1)/odds ratio, where α is the baseline rate of daily cause specific ED visits (see supplementary appendix).^40^ ^41^

We performed a series of sensitivity analyses to assess the robustness of our findings. First, we varied the key modeling parameters, including modeling exposure-response functions using a quadratic B spline with two and three internal knots and modeling the lag response function using a natural cubic B spline with three knots placed at equal intervals on the log scale of lags up to five days. Second, to assess whether our results were robust to the choice of exposure metric, we repeated the main analyses using exposure based on daily mean and minimum temperature rather than daily maximum temperature. Third, to disaggregate the potential effects of daily maximum and minimum temperatures, we refit the main analysis based on daily maximum temperature, with additional adjustment for the daily difference between maximum and minimum temperature on the same day (lag 0) modeled as a linear continuous variable. Finally, we used a more restrictive definition of heat related illness (ICD-9: 992, E900.0 or ICD-10: T67, X30).

To examine potential differences in susceptibility, we evaluated whether the association between ambient heat and risk of ED visits varied across strata defined by age, sex, low income status, NCA4 region, and Köppen-Geiger climate zone. We used the Wald test to assess whether the associations were homogeneous across strata.^42^

All analyses were conducted in R version 3.6.3. The “survival” package version 3.2-7 was used for the conditional logistic regression and the “dlnm” package version 2.4.2 was used for the distributed lag non-linear model.

### Patient and public involvement

As this study used deidentified claims data, no patient or member of the public was involved in implementing the study design. We have no plans to disseminate the results of the research directly to study participants.

## Results

Days of extreme heat were defined based on the local, county specific distribution of maximum daily temperatures during the warm season (May to September) in each location (fig 1A). Although the definition of extreme heat depends on the location, the average temperature of 34.4°C (93.9°F) was considered as extreme across the country.

Overall, 21 996 670 ED visits were recorded during the warm season between 2010 and 2019 among 74 188 445 beneficiaries residing in 2939 US counties (fig 1). Two hundred and four counties (representing about 1% (2 989 017/319 248 785) of the US population) with no recorded ED visits during the study period were excluded. On 1 July 2015, the study population consisted of 20.4 million commercially insured and Medicare Advantage beneficiaries, accounting for about 6.4% (20 437 195/319 248 785) of the US resident population in 2015. Compared with the US population, beneficiaries in the study on this date were more likely to be young and middle aged adults, men, and residing in the Midwest and southern great plains (supplementary table S2).

**Fig 2:**
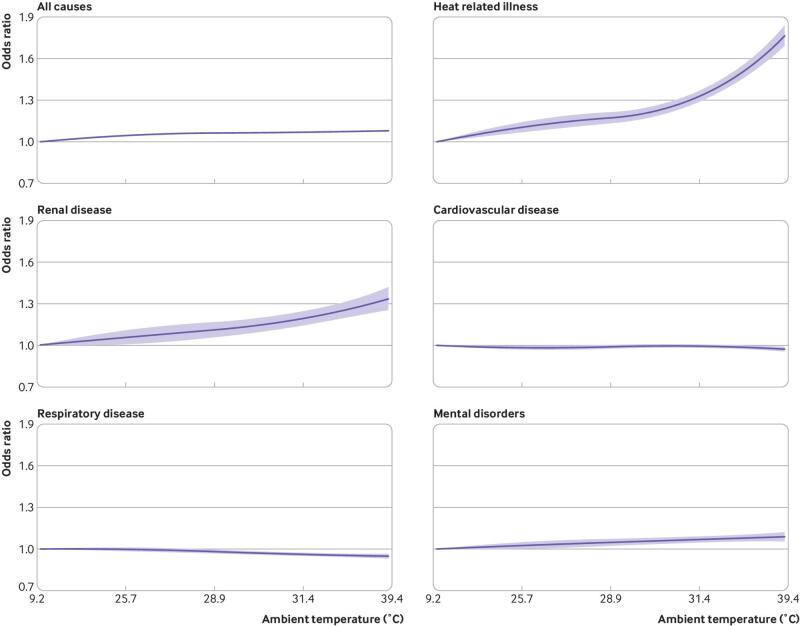
National cumulative exposure-response curves for associations between daily maximum ambient temperature and cause specific emergency department visits over lag days 0-5 in 2939 US counties, 2010-19. Solid lines represent mean odds ratios of emergency department visits (temperatures versus first centile). Shaded areas represent 95% confidence intervals. Ambient temperature (°F)=(°C×9/5)+32

**Fig 3:**
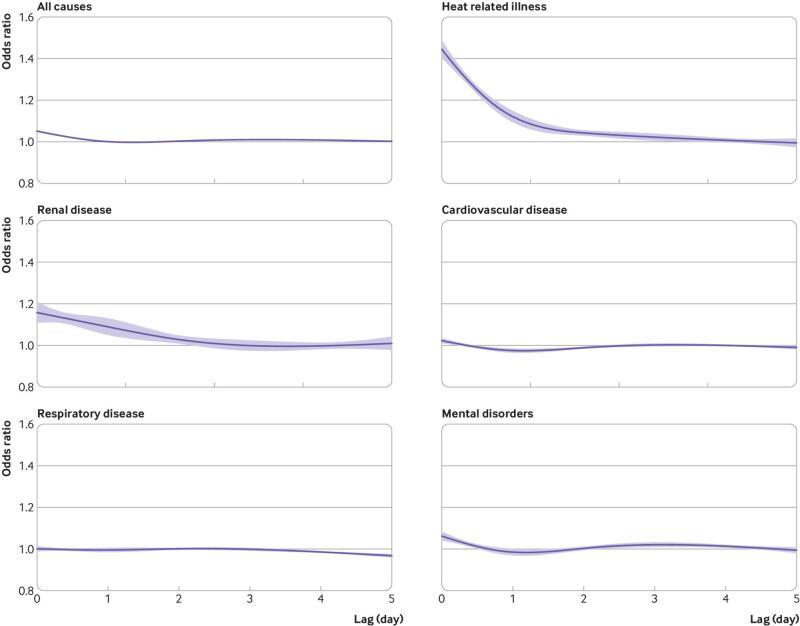
Overall lag structure in effects of extreme heat on cause specific emergency department visits in 2939 US counties, 2010-19. Extreme heat was defined based on 95th centiles of the local county specific warm season temperature distribution and excess risks are expressed versus the local first centile. On average across the country, extreme heat was at 34.4°C. Solid lines represent mean estimates. Blue bands represent 95% confidence intervals

A monotonic association was observed between daily maximum temperature and relative risk of ED visits from any cause, with no clear evidence of a threshold (fig 2). For example, a day of extreme heat was associated with a 7.8% (95% confidence interval 7.3% to 8.2%) excess relative risk of ED visits for any cause compared with the temperature of minimum morbidity defined as the local temperature corresponding to the first centile of the warm season distribution of maximum daily temperatures. Warm season temperatures were also associated with higher risks of ED visits for heat related illness, renal disease, and mental disorders (fig 2). For example, days of extreme heat were associated with a 66.3% (60.2% to 72.7%) higher relative risk of ED visits for heat related illness, 30.4% (23.4% to 37.8%) higher relative risk of ED visits for renal disease, and 7.9% (5.2% to 10.7%) higher relative risk of ED visits for mental disorders. Days of moderate heat—defined as days with a maximum temperature equivalent to the 85th centile of the local temperature distribution during the warm season—were also associated with a higher risk of ED visits for any cause and for heat related illnesses, renal disease, and mental disorders (table 1). The association between heat and risk of ED visits was most pronounced on the same day (lag 0), but with some evidence of continued higher risk over the subsequent 1-2 days (fig 3). No evidence was found of a positive association between heat and ED visits for cardiovascular or respiratory disease (fig 2).

**Table 1:** Excess relative risk and excess absolute risk of cause specific emergency department (ED) visits associated with moderate and extreme temperature over lag days 0-5 in 2939 US counties, 2010-19*

Reason for ED visits	Average moderate heat (32.6°C)			Average extreme heat (34.4°C)	
	Excess relative risk (%)	Excess absolute risk (No/100 000 people at risk/day)		Excess relative risk (%)	Excess absolute risk (No/100 000 people at risk/day)
Any cause	7.3 (6.9 to 7.6)	7.9 (7.5 to 8.3)		7.8 (7.3 to 8.2)	8.4 (7.9 to 8.8)
Heat related illness	46.6 (42.1 to 51.2)	19.3 (18.0 to 22.6)		66.3 (60.2 to 72.7)	24.3 (22.9 to 25.7)
Renal disease	24.2 (18.7 to 29.9)	12.3 (10.0 to 14.6)		30.4 (23.4 to 37.8)	14.7 (12.1 to 17.4)
Cardiovascular disease	−1.1 (−2.4 to 0.2)	−0.8 (−1.7 to 0.1)		−2.2 (−3.7 to −0.6)	−1.5 (−2.7 to −0.4)
Respiratory disease	−4.5 (−5.7 to −3.2)	−3.5 (−4.4 to −2.5)		−5.0 (−6.5 to −3.4)	−3.9 (−5.1 to −2.6)
Mental disorders	7.1 (5.0 to 9.4)	5.4 (3.9 to 7.0)		7.9 (5.2 to 10.7)	5.9 (4.0 to 7.9)
Negative control: epilepsy	−1.7 (−8.2 to 5.4)	−1.3 (−6.8 to 4.2)		−3.3 (−11.2 to 5.3)	−2.7 (−9.7 to 4.2)

Ambient temperature (°F)=(°C×9/5)+32.

* Moderate and extreme heat were defined based on the 85th and 95th centiles of local county specific temperature distribution during the warm season, and excess risks are expressed versus the local first centile.

Table 1 presents the association between days of moderate or extreme heat and the excess absolute risk of ED visits, expressed as the number of expected excess ED visits per 100 000 people at risk per day compared with the local first temperature centile. For example, days of extreme heat were associated with 8.4 (95% confidence interval 7.9 to 8.8), 24.3 (22.9 to 25.7), 14.7 (12.1 to 17.4), and 5.9 (4.0 to 7.9) excess ED visits per 100 000 people at risk each day for any cause, heat related illness, renal disease, and mental disorders, respectively. No evidence was found of an association between heat and risk of ED visits for epilepsy, an outcome selected as a putative negative control with no known association with heat. For example, days of extreme heat were associated with an excess relative risk of −3.3% (95% confidence interval −11.2% to 5.3%) and an excess absolute risk of −2.7 (−9.7 to 4.2) per 100 000 people at risk each day for epilepsy (table 1).

A series of sensitivity analyses to assess the robustness of the results showed that the results were not materially different when an alternative number of knots was used for the exposure-response function and lag-response function of temperature (supplementary figures S2 and S3). Results and conclusions were also similar when mean or minimum daily temperatures were considered as the exposure metric instead of maximum daily temperature (supplementary tables S3 and S4), or when models were additionally adjusted for daily difference between maximum and minimum temperatures (supplementary table S5). The association between temperature and heat related illness was more pronounced when an alternative, more specific, definition of heat related ED visits was used (supplementary table S6).

When the association between warm season temperatures and ED visits for any cause were evaluated within strata defined by age and sex (table 2), days of extreme heat were associated with a higher risk of all cause ED visits in every age group, with generally stronger associations evident among young and middle aged adults. For example, a day of extreme heat was associated with an excess relative risk of 3.6% (95% confidence interval 2.7% to 4.6%) among adults aged ≥75 years compared with 10.3% (9.1% to 11.5%) among those aged 45 to 54 years. The excess absolute risk associated with a day of extreme heat was 10.2 (95% confidence interval 9.0 to 11.4) excess ED visits per 100 000 people at risk per day among adults aged 25 to 34 years. Both the excess absolute and the relative risks were greater among men than among women.

**Table 2:** Excess relative risk and excess absolute risk (95% confidence interval) of emergency department (ED) visits for all causes associated with extreme heat over lag days 0-5, by age, sex, and low income status

Characteristics	No (%) of ED visits	Excess relative risk (%)	P value	Excess absolute risk (No/100 000 people at risk/day)	P value
Age (years):					
18-24	2 102 380 (9.6)	9.5 (8.0 to 11.0)	<0.001	8.5 (7.3 to 9.7)	<0.001
25-34	2 904 132 (13.2)	9.9 (8.6 to 11.1)		10.2 (9.0 to 11.4)	
35-44	2 906 914 (13.2)	7.4 (6.2 to 8.6)		6.9 (5.8 to 7.9)	
45-54	3 183 433 (14.5)	10.3 (9.1 to 11.5)		9.0 (8.0 to 9.9)	
55-64	3 276 096 (14.9)	8.8 (7.6 to 10.0)		8.0 (7.0 to 9.0)	
65-74	3 228 092 (14.7)	7.6 (6.4 to 8.7)		7.6 (6.5 to 8.8)	
≥75	4 395 623 (20.0)	3.6 (2.7 to 4.6)		4.5 (3.3 to 5.7)	
Sex:					
Men	9 314 254 (42.4)	9.5 (8.8 to 10.2)	<0.001	9.6 (8.9 to 10.2)	<0.001
Women	12 678 437 (57.6)	6.5 (5.9 to 7.1)		7.4 (6.8 to 8.0)	
Low income status*:					
Yes	3 111 751 (41.0)	5.6 (4.6 to 6.6)	0.53	12.6 (10.2 to 14.9)	<0.001
No	4 476 250 (59.0)	6.1 (4.9 to 7.3)		6.0 (5.0 to 7.0)	

Ambient temperature (°F)=(°C×9/5)+32.

Extreme heat was defined based on the 95th centiles of local county specific temperature distribution during the warm season, and excess risks are expressed versus the local first centile. On average across the country, extreme heat was 34.4°C.

* Only among a subset of members (32% (n=23 803 556/74 188 445) of total unique beneficiaries) who were enrolled in Medicare Advantage programmes that include pharmacy benefits.

In a subset of individuals (32% (n=23 803 556/74 188 445) of unique beneficiaries) enrolled in Medicare Advantage programmes (people primarily aged ≥65 years) that include pharmacy benefits, information was leveraged on qualification for prescription drug subsidies as a marker of low socioeconomic means. Although beneficiaries had a similar excess relative risk of ED visits for any cause regardless of low income status (5.6% (95% confidence interval 4.6% to 6.6%) *v* 6.1% (4.9% to 7.3%) in the low income yes *v* no categories), the excess absolute risk was substantially higher in the low income yes *v* no groups (12.6 (95% confidence interval 10.2 to 14.9) *v* 6.0 (5.0 to 7.0) per 100 000 people at risk per day in the low income yes *v* no groups) owing to the higher baseline risk among the low income group (table 2).

Whether the association between warm season temperatures and risk of ED visits for any cause varied across counties grouped either geographically or by climate region was also evaluated (table 3). US counties in the northwest or those with a continental climate had the lowest median warm season temperatures, whereas those in the southern great plains or with tropical climates had the warmest. The excess relative and absolute risks associated with days of extreme heat were greatest in counties in the north east and in the continental climate zone. The association with extreme heat was weakest among counties in the south east and was not evident among counties with a tropical climate.

**Table 3:** Excess relative risk and excess absolute risk (95% confidence interval) of emergency department visits for any cause associated with extreme heat over lag days 0-5, by US Global Change Research Program’s Fourth National Climate Assessment (NCA4) region and Köppen-Geiger climate zone

Characteristics	No of counties	Median temperature (°C)	Extreme heat* (°C)	Excess relative risk† (%)	P value	Excess absolute risk† (No/100 000 persons at risk/day)	P value
**Climate zone**							
Continental	1268	26.6	32.9	10.6 (9.8 to 11.3)	<0.001	10.5 (9.9 to 11.2)	<0.001
Temperate	1388	30.9	35.4	6.0 (5.3 to 6.6)		6.8 (6.1 to 7.4)	
Dry	278	30.3	36.7	5.8 (4.1 to 7.5)		6.0 (4.3 to 7.7)	
Tropical	5	31.8	33.6	0.6 (−0.8 to 1.9)		0.7 (−0.9 to 2.2)	
**NCA4 region**							
North east	298	26.1	31.8	12.0 (10.8 to 13.2)	<0.001	11.9 (10.8 to 12.9)	<0.001
Midwest	726	27.0	32.9	9.8 (8.9 to 10.8)		9.9 (9.0 to 10.7)	
Northern great plains	226	26.4	34.0	9.6 (5.6 to 13.7)		9.6 (5.9 to 13.3)	
South west	198	28.9	34.9	7.2 (5.6 to 8.8)		7.0 (5.5 to 8.4)	
Southern great plains	401	32.7	38.5	6.8 (5.5 to 8.2)		7.4 (6.0 to 8.7)	
North west	113	24.8	33.3	5.9 (2.4 to 9.5)		6.4 (2.8 to 10.1)	
South east	977	30.8	34.8	4.3 (3.6 to 5.1)		5.3 (4.4 to 6.2)	

On average across the US, extreme heat was at 34.4°C. Ambient temperature (°F)=(°C×9/5)+32.

* Defined based on 95th centiles of the local county specific temperature distribution during the warm season.

† Excess risks are expressed versus the local first centile.

## Discussion

We conducted a nationwide study to estimate the risk of ED visits for any cause and cause specific disorders associated with temperatures during the warm season among more than 74 million adults aged 18 years and older residing in 2939 counties across the conterminous US. We found a monotonic association between warm season temperatures and risk of ED visits for any cause, as well as for heat related illnesses, renal disease, and mental disorders, without any evidence of a discernible threshold. These associations were robust to varying modeling choices and exposure metrics. The excess absolute and relative risks varied substantially and statistically significantly across strata defined by geographic region, climate zone, age, sex, and low income status (an indicator of financial need), with the strongest associations observed in the north east of the US, in counties with a continental climate, and among men, young and middle aged adults, and those receiving financial subsidies for prescription drugs.

### Comparison with other studies

Our results are consistent with a robust body of existing evidence indicating that heat increases the risk of morbidity and mortality, that this excess risk is observed across a range of warm season temperatures with no clear threshold, that the strongest associations are observed on the same day as the raised temperatures, and that the degree of excess risk varies across individuals and communities.^6^ ^8^ ^9^ ^13^ ^14^ ^15^ ^16^ ^17^ ^24^ ^25^ ^43^ ^44^ However, much of the previous evidence has been derived from studies of the impacts of heat either on mortality or on hospital admissions among elderly people,^8^ ^13^ ^14^ ^15^ ^16^ ^17^ with few large scale studies examining the impacts of heat on adults across the full age range or on ED visits rather than hospital admissions. The results of the present study extend this previous knowledge and indicate that the adverse health impacts of heat are at least as important among young and middle aged adults as among elderly people, in terms of both relative and absolute risks. In addition, these results show that ED visits can serve as a sensitive and timely marker of the adverse health impacts of heat across the age spectrum.

Although our results are consistent with the previous literature, it is difficult to directly compare effect estimates across studies given the use of different exposure metrics, exposure contrasts, time periods, study populations, and analytic methods. For example, an analysis of US Medicare beneficiaries aged 65 years and older in 114 cities between 1992 and 2006 found that days of extreme heat (defined as a day with warm season apparent temperature at the 99th city specific temperature centile *v* 75th centile) were associated with a 3.2% (95% confidence interval 2.4% to 4.0%) higher risk of emergency hospital admissions for any cause.^14^ It is difficult to compare this result with the result from our study, given, for example, the difference in exposure metric (apparent temperature *v* ambient temperature), the difference in outcome (hospital admission *v* ED visit), and the time period. In another study in California, the authors found that the heatwave period (defined as the dates of the first and the last reported heat related deaths) was associated with a higher risk of ED visits for any cause, with an excess relative risk of 3% (95% confidence interval 2% to 4%).^18^ The results are also not comparable with ours, primarily because of the difference in heat exposure (heatwave period *v* county specific 95th temperature centile).

Our findings of no evidence of a positive association between heat and ED visits for cardiovascular and respiratory diseases were also consistent with most previous studies.^14^ ^18^ ^43^ For example, in an analysis of hospital admissions in 12 European cities, the authors reported that the association between high temperature and cardiovascular admissions tended to be negative and did not reach statistical significance.^9^ Some have suggested that these results could indicate that during extreme heat events, people are more likely to die before being admitted to a hospital.^9^ ^45^ This explanation is supported by previous reports of a greater burden of dying outside a hospital than inside a hospital during extreme heat events.^45^ ^46^

Previous research has established that some individuals or populations are at much greater risk of heat related health effects than others, although which groups are identified as most susceptible varies across studies. We found that the effects of heat on ED visits for any cause are more pronounced among young and middle aged adults than among older adults. Published studies on susceptible populations by age are mixed, with some finding stronger associations among older adults than among younger adults,^43^ ^44^ and others finding the reverse.^6^ ^24^ ^25^ ^47^ Our results are consistent with analyses of data from the US state of Rhode Island,^6^ 18 sites in China,^24^ and Vietnam,^47^ which found a more pronounced association of heat with ED visits among young and middle aged adults compared with older adults. For example, a study in Rhode Island among 0.5 million heat related ED admissions reported that an increase in daily maximum temperature from 26.7°C to 32.2°C was associated with a 59.6% (95% confidence interval 44.7% to 76.0%) higher risk of heat related ED visits for adults aged 18-64 years and 22.6% (11.8% to 34.3%) for those aged 65 years and older.^6^ One possible explanation for these results could be that adults of working age are more likely to have occupational and recreational activities that increase opportunities for exposure to heat. Alternatively, increased public awareness of heat related health risks among elderly people and public health efforts to reduce these dangers might be effective at reducing risk in this population.^48^

Our findings that the adverse health effects of heat are more pronounced among men than among women is also consistent with findings from studies of hospital admissions among US Medicare beneficiaries,^17^ and studies from other countries.^49^ ^50^ Most previous studies find that the adverse health impacts of heat are more pronounced among people of lower socioeconomic means.^17^ ^21^ ^22^ In a subset of the population, we found that the association between heat and ED visits for all causes was similar in relative terms among those with compared with those without a marker of low socioeconomic means. However, the excess absolute risk differed between the groups given that the baseline rate of ED visits was higher for those with the marker of prescription drug subsidy.

Noticeable geographic differences in vulnerability to extreme heat have been well documented, even in studies where heat is defined relative to local area norms.^8^ ^15^ For example, people in locations with cooler climates or with a lower prevalence of air conditioning, or both, have typically been found to have higher vulnerability to heat.^8^ ^15^ We similarly found that the associations with heat were more noticeable among residents of US counties in the north east, Midwest, or with continental climates and relatively weaker in counties in the south east or with tropical climates. These results are consistent with a hypothesized lesser degree of heat adaptation in areas with colder climates, which might be related to physiological or behavioral factors, such as less availability of air conditioning in areas with colder climates.^17^

Compared with mortality and hospital admissions, relatively fewer studies have examined ED visits as a marker of the adverse health impacts of heat. ED visits are thought to be a sensitive indicator of trends for diseases affected by heat^18^ and are likely a more appropriate setting for both public health surveillance^10^ and tertiary prevention. For example, during the heatwave in California in July 2006, the number of excess ED visits attributable to heat were about 13-fold larger than the excess number of hospital admissions attributed to heat (16 166 *v* 1182).

### Limitations and strengths of this study

This study has several potential limitations. First, we used the population weighted average of daily maximum temperature in each county as a proxy for personal heat exposure. Some amount of exposure misclassification is inevitable because of uncertainty in the location and time activity pattern of any individual in the study, a limitation shared by all time series studies and likely to lead to an underestimation of the associations or reduction in statistical power, or both.^51^ Exposure misclassification might, however, have been lower in this study compared with many previous studies given our use of ambient temperature estimated from a spatially refined, gridded climate dataset rather than data from airport weather stations, which might not represent population average exposures.^33^ Second, for most patients we only have individual information on age and sex and do not have detailed information on other patient characteristics, such as race, occupation, health behaviors, socioeconomic means, access to air conditioning, or time activity patterns. Thus, we were not able to assess whether the impacts of heat differed across these characteristics. Third, our study population included only US adults with health insurance, a population that might be healthier, of higher socioeconomic means, or otherwise potentially less susceptible to the adverse health effects of heat compared with the general population. We expect that the estimated associations might be even more noticeable among people without commercial health insurance. Indeed, our results might not be generalizable to people without health insurance, to children and adolescents, to those living in counties within the conterminous US where we did not have data (in which about 1% of the US population live), or to locations outside of the conterminous US.

One strength of our study was the large sample size—more than 22 million ED visits among enrollees of commercial and Medicare Advantage health insurance plans aged 18 years or older across different geographic regions and climate zones in the conterminous US. We were therefore able to comprehensively analyze daily ambient temperature and risk of cause specific ED visits among this large sample.

### Policy implications

Days of extreme heat are a recognized public health problem associated with high morbidity and mortality. The present study adds to the existing literature on the health effects of heat by showing that adults of all ages are at increased risk of heat related health effects rather than just elderly people, providing estimates of the potential impact of heat in terms of both excess absolute and relative risk, and documenting that the risk of heat associated illness is apparent across every region of the conterminous US, particularly for regions with cooler climates.

The adverse health impacts of extreme heat are thought to be largely preventable through any combination of reduced exposure, reduced susceptibility, or improved adaptive capacity. For example, existing heat early warning and response systems typically include dissemination of information to the public and key stakeholders, facilitating coordination among local agencies, opening designated cooling centers, and other strategies for communicating and reducing health risks.^52^ However, a fundamental premise of disaster or emergency preparedness is that the response in any given location should be informed by local factors, including an assessment of the expected local impacts of hazards, and local assessments of potential exposures, vulnerabilities, and adaptive capacity.^52^ In the context of public health preparedness for days of extreme heat, it is essential that communities and community leaders understand local risks posed by specific locally defined temperature thresholds.^52^ For example, a day with a maximum temperature of 35°C might be rare and dangerously hot in one community and more common or not highly dangerous in a different community. Indeed, the maximum daily temperature considered as extreme varies substantially across the US (fig 1). Moreover, we found that the excess relative and absolute risk of ED visits for heat related illness on days of locally extreme heat varied substantially by location, thus highlighting the importance of integrating existing evidence from population scale studies such as this one with local knowledge and assessments to guide locally appropriate heat early warning and response systems. It is also important for hospitals and other facilities to adapt their procedures to meet the increased demand that extreme heat can place on local healthcare systems.

### Conclusions

Among US adults with health insurance, days of extreme heat were associated with a higher relative risk of ED visits for any cause, heat related illness, renal disease, and mental disorders. Although everyone is at risk of the adverse health impacts of heat, some individuals and some communities are more noticeably at greater risk than others. This information might be useful to clinicians, public health officials, and the public considering the potential for more frequent and severe extreme heat events attributable to the rapidly changing climate.

**What is already known on this topic**

Days of extreme heat are associated with an increased risk of deaths and hospital admissions among older adults (age ≥65 years)
Less is known about the adverse health impacts of heat among young and middle aged adults
Emergency department (ED) visits might provide a more sensitive marker of the adverse health impacts of heat versus hospital admissions, especially in younger adults

**What this study adds**

In this nationwide study in the US, days of extreme heat were associated with a higher risk of ED visits for any cause, heat related illness, renal disease, and mental disorders
The adverse health effects of extreme heat are not limited to older adults, with important excess risk observed in both young and middle aged adults
The adverse health impacts of heat varied among individuals (with men and low income adults at greatest risk) and across communities (with those in the US counties in the north east or with continental climates at greatest risk)

## Data Availability

No additional data available.

## Web extra

Extra material supplied by authors

Supplementary information: appendices, tables S1-6, figures S1-3, and relevant references

## Notes

Contributors: SS, KRW, GAW, and FD designed the study. SS and GAW developed the analysis plan. SS and YS performed statistical analyses and took responsibility for the accuracy of the data analysis. SS and GAW drafted the manuscript. All authors contributed to interpretation of the results and the revision and approval of the final manuscript. SS is the guarantor. The corresponding author attests that all listed authors meet authorship criteria and that no others meeting the criteria have been omitted.

Funding: This study is supported by grants R01-ES029950 (SS, ANS, KRS, YS, FD, GAW) and F32-ES027742 (KRW) from the US National Institutes of Health/National Institute of Environmental Health Sciences, and grant 216033-Z-19-Z from the Wellcome Trust (SS, ANS, KRS, GAW). The funders had no role in considering the study design or in the collection, analysis, interpretation of data, writing of the report, or decision to submit the article for publication.

The corresponding author (SS) affirms that the manuscript is an honest, accurate, and transparent account of the study being reported; that no important aspects of the study have been omitted; and that any discrepancies from the study as planned and registered have been explained.

Dissemination to participants and related patient and public communities: There are no plans to disseminate the results of the research directly to study participants. We plan to disseminate the results of the research to the general public through media outreach—for example, press releases by the media departments of the authors’ research institutes, and plain language publications in social media.

Publisher’s note: Published maps are provided without any warranty of any kind, either express or implied. BMJ remains neutral with regard to jurisdictional claims in published maps.

Provenance and peer review: Not commissioned; externally peer reviewed.
