# Supplementary material for: Ambient heat and risks of emergency department visits among adults in the United States: time stratified case crossover study
Source: BMJ. 2021 Nov 25;375:e065653. doi: 10.1136/bmj-2021-065653 (PMC9397126; doi:10.1136/bmj-2021-065653)
Supplement: Supplementary file 1 — Supplementary information: appendices, tables S1-6, figures S1-3, and relevant references [file suns065653.ww.pdf]

<u>Supplementary Online Content</u>

Ambient Heat and Risks of Emergency Department Visits among Adults in the United States

Shengzhi Sun, Kate R. Weinberger, Amruta Nori-Sarma, Keith R. Spangler, Yuantong Sun, Francesca Dominici, Gregory A. Wellenius

Table of Contents

eAppendix. Ambient temperature assessment

eAppendix. Calculation of absolute excess risk

Table S1. Codes to identify emergency department visits.

Table S2. The characteristics of the study beneficiaries and US resident population on 1 July 2015.

Table S3. Excess relative risk and excess absolute risk of cause-specific emergency department visits associated with moderate and extreme temperature defined by daily mean temperature over lag days 0-5 in 2,939 US counties, 2010-2019.

Table S4. Excess relative risk and excess absolute risk of cause-specific emergency department visits associated with moderate and extreme temperature defined by daily minimum temperature over lag days 0-5 in 2,939 US counties, 2010-2019.

Table S5. Excess relative risk of cause-specific emergency department visits associated with extreme temperature defined by daily maximum temperature over the study period over lag days 0-5 in 2,939 US counties, 2010-2019, results from main analysis compared with a model additionally adjusted for daily difference between maximum and minimum temperature on the same day (lag 0) modeled as a linear continuous variable.

Table S6. Relative risk and excess absolute risk of heat-related emergency department visits associated with moderate and extreme temperature defined by daily maximum temperature over lag days 0-5 in 2,939 US counties, 2010-2019.

Figure S1. Köppen-Geiger climate zone (A) and Global Change Research Program's Fourth National Climate Assessment geographic region (B) and in the United States.

Figure S2. Sensitivity analysis by testing different modelling choices for cumulative exposure-response curves for the association between daily maximum temperature and emergency department visits for any cause, heat-related illness, renal, cardiovascular, respiratory disease, and mental disorders.

Figure S3. Sensitivity analysis by testing different modelling choices for overall lag structure in effects of extreme heat on emergency department visits for any cause, heat-related illness, renal, cardiovascular, respiratory disease, and mental disorders.

Supplemental references

eAppendix. Ambient Temperature Assessment

We estimated daily maximum ambient temperature using the Parameter-elevation Relationships on Independent Slopes (PRISM) model, a validated spatiotemporal model with approximately four-kilometer horizontal grid spacing.[1] To represent population exposure to temperature, we calculated a population-weighted average of daily maximum temperature for each day in each county.[2] Briefly, we first obtained the population centroids for each census tract in each of the 2,939 US counties of interest from the 2000 Census. Next, we extracted daily PRISM-predicted temperatures at the grid cell overlaying each of these census tract population centroids. Finally, we used these extracted grid cells to calculate a daily time series of population-weighted maximum temperature for each day in each county. Specifically, for each extracted grid cell, we multiplied its daily temperature value by the proportion of the county population falling within that census tract. We then summed the resulting values across all grid cells located within each county to obtain the county population-weighted mean value.

Appendix. Calculation of absolute excess risk

To calculate the absolute excess risk in ED visits associated with heat, we used $\alpha_c(1 - \exp(-\widehat{\beta_c}))$ with a standard error calculated as $\alpha_c \exp(-\widehat{\beta_c})\, \widehat{se_c}$, where α_c is the baseline rate for cause-specific ED visits c, calculated using the total number of cause-specific ED visits during 2010 to 2019 among beneficiaries covered by a health plan divided by the total person-days for beneficiaries covered by a health plan, and $\widehat{\beta_c}$ and $\widehat{se_c}$, are the coefficient and standard error for heat extracted from the conditional logistic regression for cause-specific ED visits c.[3,4]

Table S1. Codes to identify emergency department visits.

Current Procedural Terminology (CPT) codes	99281, 99282, 99283, 99284, 99285, 99288
Revenue code	0450, 0451, 0452, 0453, 0454, 0455, 0456, 0457, 0458, 0459, 0981
Place of service code	23

Table S2. The characteristics of the study beneficiaries and US resident population on 1 July 2015.

Characteristics	No. of enrollees on 1 July 2015	No. of resident population on 1 July 2015[a]	% of enrollees	*p*-value[b]
Total No.	20,437,195 (100.0)	319,248,785 (100.0)	6.4	--
Age, years				<0.001
<20	4,461,357 (21.8)	81,578,778 (25.6)	5.5	
20-64	12,893,647 (63.1)	190,218,906 (59.6)	6.8	
≥65	3,082,191 (15.1)	47,451,101 (14.9)	6.5	
Gender				<0.001
Male	10,434,342 (51.1)	157,114,900 (49.2)	6.6	
Female	10,002,853 (48.9)	162,133,885 (50.8)	6.2	
NCA4 Region				<0.001
Northeast	3,449,765 (16.9)	65,752,582 (20.6)	5.2	
Southeast	5,263,111 (25.8)	80,333,704 (25.2)	6.6	
Midwest	5,015,397 (24.5)	61,484,176 (19.3)	6.2	
Northern Great Plains	322,126 (1.6)	5,130,642 (1.6)	6.3	
Northwest	485,123 (2.4)	12,854,258 (4.0)	3.8	
Southern Great Plains	3,082,258 (15.1)	34,292,093 (10.7)	9.0	
Southwest	2,819,415 (13.8)	59,401,330 (18.6)	4.7	

Abbreviations: NCA4, US Global Change Research Program's Fourth National Climate Assessment

--Not applicable.

[a]Number of US resident population were obtained from the US Census Bureau, Population Division.[5]

[b]p-values were calculated using Chi-squared test.

Table S3. Excess relative risk and excess absolute risk of cause-specific emergency department visits associated with moderate and extreme temperature defined by daily mean temperature over lag days 0-5 in 2,939 US counties, 2010-2019. Moderate and extreme heat were defined based on the 85th and 95th percentiles of the local county-specific warm-season temperature distribution and excess risks are expressed versus the local 1st percentile. On average across the country, moderate heat was at 26.0 ºC and extreme heat was at 27.5 ºC. Data presented as mean and 95% confidence intervals. Ambient temperature (°F) = (°C×9/5) + 32.

Disease	Moderate heat (26.0 ºC on average)		Extreme heat (27.5 ºC on average)	
	Excess relative risk (%)	Excess absolute risk (No. per 100,000 persons at risk per day)	Excess relative risk (%)	Excess absolute risk (No. per 100,000 persons at risk per day)
All-cause	7.3 (7.0 to 7.7)	7.9 (7.6 to 8.3)	7.6 (7.2 to 8.1)	8.2 (7.8 to 8.7)
Heat-related	48.7 (44.2 to 53.3)	19.9 (18.7 to 21.2)	68.7 (62.7 to 75.0)	24.8 (23.5 to 26.1)
Renal disease	24.1 (18.7 to 29.7)	12.2 (10.0 to 14.5)	31.6 (24.7 to 38.9)	15.2 (12.6 to 17.7)
Cardiovascular	-1.2 (-2.4 to 0.10)	-0.83 (-1.73 to 0.06)	-2.3 (-3.8 to -0.71)	-1.6 (-2.7 to -0.49)
Respiratory	-5.9 (-7.0 to -4.7)	-4.6 (-5.6 to -3.6)	-6.9 (-8.3 to -5.4)	-5.4 (-6.7 to -4.2)
Mental disorders	7.7 (5.5 to 9.9)	5.8 (4.3 to 7.3)	8.9 (6.3 to 11.7)	6.7 (4.8 to 8.5)
Negative control: Epilepsy	0.10 (-6.4 to 7.1)	0.07 (-5.2 to 5.4)	-1.2 (-9.1 to 7.3)	-0.10 (-7.6 to 5.6)

Table S4. Excess relative risk and excess absolute risk of cause-specific emergency department visits associated with moderate and extreme temperature defined by daily minimum temperature over lag days 0-5 in 2,939 US counties, 2010-2019. Moderate and extreme heat were defined based on the 85th and 95th percentiles of the local county-specific warm-season temperature distribution and excess risks are expressed versus the local 1st percentile. On average across the country, moderate heat was at 19.9 ºC and extreme heat was at 21.3 ºC. Data presented as mean and 95% confidence intervals. Ambient temperature (°F) = (°C×9/5) + 32.

**Disease**	**Moderate heat (19.9 ºC on average)**		**Extreme heat (21.3 ºC on average)**	
	**Excess relative risk (%)**	**Excess absolute risk (No. per 100,000 persons at risk per day)**	**Excess relative risk (%)**	**Excess absolute risk (No. per 100,000 persons at risk per day)**
All-cause	7.1 (6.8 to 7.5)	7.7 (7.4 to 8.1)	7.4 (7.0 to 7.8)	8.0 (7.6 to 8.5)
Heat-related	49.0 (44.5 to 53.6)	20.0 (18.8 to 21.3)	65.0 (59.1 to 71.1)	24.0 (22.6 to 25.3)
Renal disease	23.3 (18.0 to 28.9)	11.9 (9.7 to 14.2)	29.9 (23.1 to 37.1)	14.5 (11.9 to 17.2)
Cardiovascular	-0.82 (-2.1 to 0.44)	-0.58 (-1.5 to 0.31)	-1.5 (-3.0, 0.10)	-1.0 (-2.1 to 0.08)
Respiratory	-6.7 (-7.8 to -5.5)	-5.3 (-6.3 to -4.3)	-8.0 (-9.4 to -6.5)	-6.4 (-7.6 to -5.1)
Mental disorders	7.7 (5.6 to 9.9)	5.8 (4.3 to 7.3)	9.1 (6.5 to 11.9)	6.8 (5.0 to 8.6)
Negative control: Epilepsy	0.53 (-6.0 to 7.5)	0.41 (-4.9 to 5.7)	-0.43 (-8.3 to 8.2)	-0.34 (-6.9 to 6.2)

Table S5. Excess relative risk of cause-specific emergency department visits associated with extreme temperature defined by daily maximum temperature over the study period over lag days 0-5 in 2,939 US counties, 2010-2019, results from main analysis compared with a model additionally adjusted for daily difference between maximum and minimum temperature on the same day (lag 0) modeled as a linear continuous variable. Extreme heat was defined based on the 95th percentiles of the local county-specific warm-season temperature distribution and excess risks are expressed versus the local 1st percentile. Abbreviation: ED visit=emergency department visit.

	Main Model	Sensitivity analysis	
**Cause of ED Visit**	**Excess Relative Risk (%) associated with maximum daily temperature at 95th percentile**	**Excess Relative Risk (%) associated with maximum daily temperature at 95th percentile**	**Excess Relative Risk (%) associated with a 10°C increase in the difference between daily maximum and minimum temperature**
All-cause	7.8 (7.3 to 8.2)	8.0 (7.5 to 8.4)	-0.8 (-1.1 to -0.5)
Heat-related	66.3 (60.2 to 72.7)	67.3 (61.0 to 73.8)	-2.3 (-4.8 to 0.2)
Renal disease	30.4 (23.4 to 37.8)	30.9 (23.8 to 38.5)	-1.5 (-5.1 to 2.1)
Cardiovascular	-2.2 (-3.7 to -0.6)	-2.2 (-3.8, -0.6)	0.3 (-0.8 to 1.3)
Respiratory	-5.0 (-6.5 to -3.4)	-5.4 (-6.9 to -3.8)	0.7 (-0.6 to 2.0)
Mental disorders	7.9 (5.2 to 10.7)	8.1 (5.3 to 10.9)	-0.6 (-2.2 to 1.0)
Negative control: Epilepsy	-3.3 (-11.2 to 5.3)	-3.5 (-11.6 to 5.2)	0.8 (-4.9 to 6.4)

Table S6. Relative risk and excess absolute risk of heat-related emergency department visits associated with moderate and extreme temperature defined by daily maximum temperature over lag days 0-5 in 2,939 US counties, 2010-2019. Moderate and extreme heat were defined based on the 85th and 95th percentiles of the local county-specific warm-season temperature distribution and excess risks are expressed versus the local 1st percentile. On average across the country, moderate heat was at 32.6 ºC and extreme heat was at 34.4 ºC. Data presented as mean and 95% confidence intervals. Ambient temperature (°F) = (°C×9/5) + 32.

**ICD codes**	**Moderate heat (32.6 ºC on average)**		**Extreme heat (34.4 ºC on average)**	
	**Odds ratio**	**Excess absolute risk (No. per 100,000 persons at risk per day)**	**Odds ratio**	**Excess absolute risk (No. per 100,000 persons at risk per day)**
Original definition: ICD-9: 276, 992, E900.0, and E900.9 or ICD-10: T67, E86, E87, X30	1.49 (1.45 to 1.53)	20.0 (18.8 to 21.3)	1.65 (1.59 to 1.71)	24.0 (22.6 to 25.3)
Restrictive definition: ICD-9: 992, E900.0 or ICD-10: T67, X30	28.3 (23.3 to 34.5)	56.1 (55.7 to 56.5)	52.4 (42.4 to 64.8)	57.1 (56.8 to 57.3)

Figure S1. Köppen-Geiger climate zone (A) and Global Change Research Program's Fourth National Climate Assessment geographic region (B) and in the United States.

A

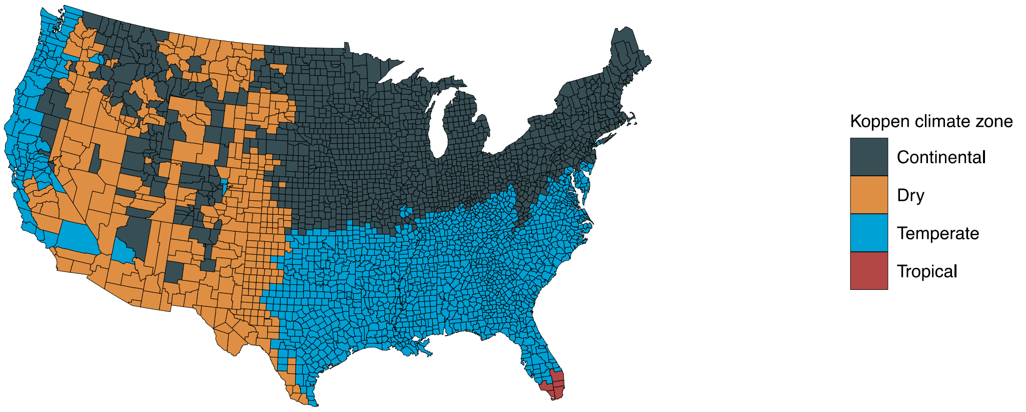

B

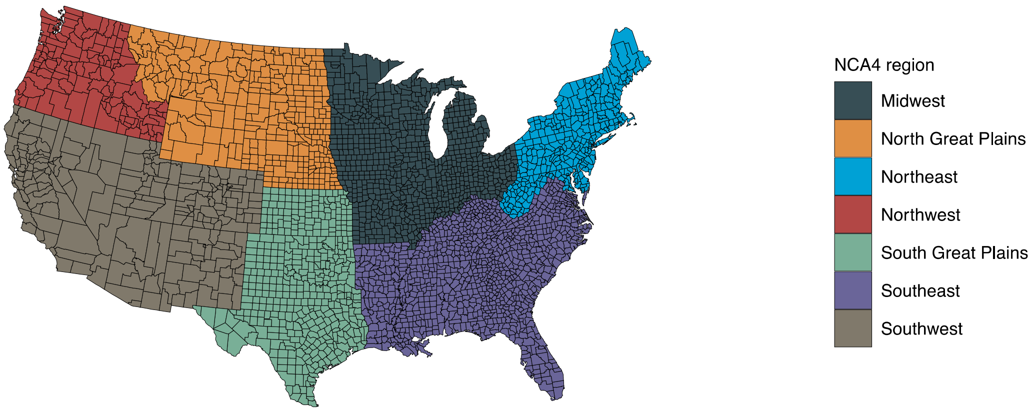

Figure S2. Sensitivity analysis by testing different modelling choices for cumulative exposure-response curves for the associations between daily maximum temperature and emergency department visits for any cause, heat-related illness, renal, cardiovascular, respiratory disease, and mental disorders. The blue bands represent 95% confidence intervals of the main model.

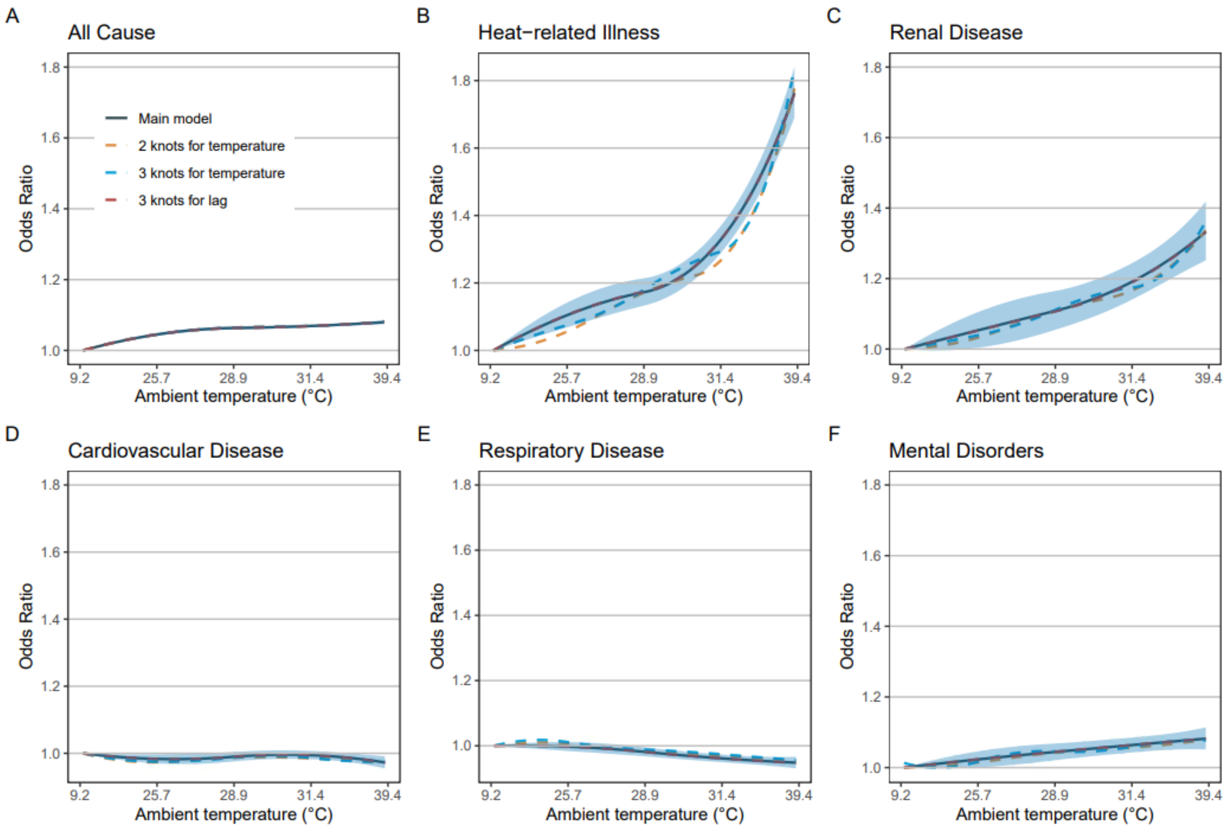

Figure S3. Sensitivity analysis by testing different modelling choices for overall lag structure in effects of extreme heat on emergency department visits for any cause, heat-related illness, renal, cardiovascular, respiratory disease, and mental disorders. The blue bands represent 95% confidence intervals of the main model.

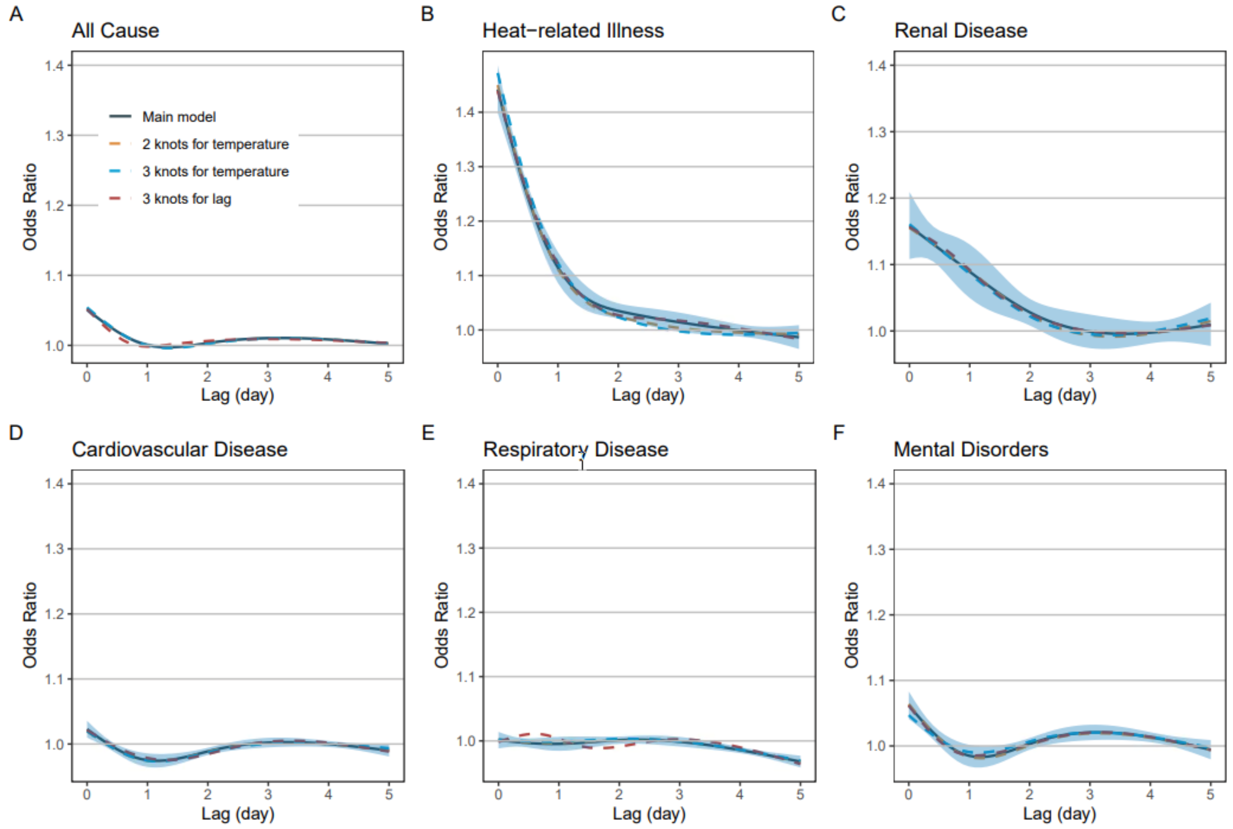

Supplemental references

1. Daly C, Halbleib M, Smith JI, et al. Physiographically sensitive mapping of climatological temperature and precipitation across the conterminous United States. *Int J Climatol.* 2008;28(15):2031-2064.
2. Spangler KR, Weinberger KR, Wellenius GA. Suitability of gridded climate datasets for use in environmental epidemiology. *J Expo Sci Environ Epidemiol.* 2019;29(6):777-789.
3. Wei Y, Wang Y, Di Q, et al. Short term exposure to fine particulate matter and hospital admission risks and costs in the Medicare population: time stratified, case crossover study. *bmj.* 2019;367.
4. Di Q, Dai L, Wang Y, et al. Association of short-term exposure to air pollution with mortality in older adults. *Jama.* 2017;318(24):2446-2456.
5. US Census Bureau. County Population by Characteristics: 2010-2019. https://www.census.gov/data/tables/time-series/demo/popest/2010s-counties-detail.html. Published 2021. Accessed 3 March 2021, 2020.
